# SDOH Data as Health Care Data: The Multifaceted, Evolving, and Consequential Role of Law and Policy

**DOI:** 10.63116/AH.000000006

**Published:** 2026-09-01

**Authors:** Rachel Landauer, Erika Hanson, Kathryn Garfield, Sara Raza, Carmel Shachar

**Affiliations:** 1 Center for Health Law and Policy Innovation, Harvard Law School; 2 University of Washington School of Law

**Keywords:** social determinants of health, health equity, privacy, advanced analytics, information exchange infrastructure

## Abstract

**Background:**

Social determinants of health (SDOH) are increasingly recognized as vital factors shaping health outcomes in the United States. With escalating integration of SDOH data into health information platforms, questions arise about appropriate regulation to both enable data-driven care and protect against potential harms. This article reviews the current regulatory landscape governing SDOH data within the US health care system, with an emphasis on identifying critical tensions, policy strategies, and gaps salient for policymakers, practitioners, and researchers.

**Methods:**

We conducted a scan to identify federal and state regulatory activity pertaining to SDOH data within the health care contexts. We synthesized these sources to map the current landscape and tease out emerging themes.

**Results/Discussion:**

We identified 3 core areas of relevant regulation: (1) data standardization, interoperability, and exchange; (2) information privacy and security; and (3) oversight of advanced analytics, including artificial intelligence applications. Authorities actively wrestle with several key regulatory design questions, including: (1) whether to treat SDOH data created, collected, maintained, and used in or by health care settings in the same way as any other type of health information; (2) effective levers to promote desired policy outcomes; and (3) the rightsized jurisdictional balance between federal and state enforcement. Variations in decisions at these crossroads are entrenching the health care system in a scenario in which SDOH data exchange is more clearly part of the new standard of care in some states than others, and in which SDOH data is more protected in some states than others.

**Conclusion:**

Findings speak to the importance of cultivating a regulatory environment for SDOH data within the health care sector that is not only responsive to the current realities of the field but also responsive to rapid evolution and resilient to future challenges.

## Introduction

Research over the last decade demonstrates that health outcomes are determined by more than clinical encounters; in fact, research suggests that only about 20% of a person’s health outcomes are attributable to clinical care, with social determinants of health (SDOH) determining as much as 50%.^1^ It is within this context that SDOH data—that is, data aimed at tracking the conditions that comprise SDOH, such as economic stability, neighborhood and built environment, and social and community context—is becoming more important to health care at the individual, family, and community levels.^2^ Common uses of SDOH data in health care include the following:

- *Adjustments to clinical care:* Social risk information supports personalized clinical care by informing prevention, treatment, and surveillance modifications. In one study of clinicians at community health centers, researchers found that SDOH information influenced care in approximately 35% of surveyed encounters.^3^ A specific example of “social risk-informed care” is the provision of contextually sensitive nutrition counseling to a food-insecure patient with diabetes.^4^
- *Social risk intervention:* Health care organizations use SDOH data to design and implement relevant social care interventions (eg, to refer patients to community-based resources, to enroll members in responsive services and supports, and other related activities).^5^ SDOH data also inform community-level interventions conducted as part of philanthropic investments, nonprofit hospital community benefits, research, and other initiatives.^5^
- *Reimbursement rate setting:* Since 2024, the Centers for Medicare and Medicaid Services (CMS) has recognized homelessness as a “complication or comorbidity” that increases Medicare reimbursement rates for acute inpatient hospital settings.^6^ Some states have also experimented with adjusting Medicaid capitation rates based on specified social risks.^7^

Delivery system and payment policy reforms are also increasingly pushing health care organizations to collect and use SDOH data. To varying degrees of durability, CMS has experimented with mandating or otherwise incentivizing (eg, through reimbursement) SDOH screening in a broad range of settings.^8^ In multiple insurance programs, opportunities to design health-related social need (HRSN) benefits—services that respond to unmet needs created by SDOH conditions—and offer nutrition, housing, and other such services and supports to beneficiaries have been expanding. Relevant Medicaid authorities include Section 1115 demonstration waivers, managed care in lieu of services and settings, state plan authorities, and Section 1915 home and community-based services authorities.^9^^,^^10^ In Medicare Advantage, Special Supplemental Benefits for the Chronically Ill is an especially pertinent flexibility (see 42 C.F.R. § 422.102). In addition, in 2024, CMS introduced Community Health Integration services, which are personalized services to address unmet HRSN in Medicare Part B.^11^

Owing to the aforementioned factors, SDOH data are becoming increasingly captured in electronic health records (EHRs), health information exchanges (HIE), care coordination platforms, payer databases, and patient applications. Furthermore, both the free flow of that information between systems (eg, from a carceral institution to a Federally Qualified Health Center as part of Section 1115 Medicaid waiver demonstrations targeting the reentry population)^12^ and safeguards to push back against potentially harmful applications thereof (eg, access to immigration information by government entities for purposes other than health care delivery)^13^ are becoming more pressing policy priorities.

Recognizing the rapidly expanding quantity of SDOH data that is created, used, and exchanged within the US health care system and the need for appropriate protections, this paper integrates 2 arenas that have each been examined extensively but rarely in relation to one another—health information and SDOH—to reconceptualize policy at the intersection. We build on existing literature in several key ways. First, we synthesize a rapidly evolving and fragmented regulatory landscape to address a knowledge gap in how, both substantively and procedurally, SDOH data, as health care data, are regulated. Second, we surface 3 core policy design questions that, taken together, are consequential in influencing the emerging standard of care: Whether SDOH data should be treated as exceptional or as health information like any other; which regulatory levers are most promising for shaping practice; and how authority should be distributed between potential decision-makers. Finally, we translate these insights into practical implications for regulators and other stakeholders.

## Methods

We conducted a narrative legal and policy scan to identify federal and state activity relevant to the creation, use, exchange, and governance of SDOH data within the US health care system. Our goal was to map the range of emerging approaches rather than generate a comprehensive or quantitative inventory.

### Search Strategy

We used Google to identify trending topics, relevant vocabulary, and open policy questions as they are discussed in practice. We relied on government websites and legal databases as authoritative sources of binding and proposed law and policy. At the federal level, Google searches used combinations of terms such as “SDOH data,” “SDOH information exchange,” “HRSNs regulation,” “closed-loop referral privacy,” and “community information exchange infrastructure.” We also searched the Federal Register and key federal agency websites using similar keywords. At the state level, we used LegiScan to search across states for relevant bills and enacted laws. We supplemented this search by using Google to locate state agency guidance, reports, and procurement documents using state names in combination with the keywords mentioned earlier. Searches were iterative. We refined our search terms and sources as we identified new policies of interest and stopped when additional searches were no longer yielding substantively new models.

### Analysis

A broad range of types of policies were reviewed to support our scoping of the landscape, including policies that have been codified in law or regulation; proposed policies that illuminate regulatory intent or emerging directions; preambles and responses to comments; publicly available guidance that clarify or interpret regulations; and gray literature such as toolkits and reports. We prioritized materials that spoke directly to SDOH data as health information or shed light on SDOH-specific considerations. Materials must have been published before June 2025.

### Limitations

Potential limitations of our approach should be acknowledged. First, the regulatory landscapes surrounding both SDOH policy and health information policy are rapidly evolving. Relevant developments may have occurred after our search window closed. Second, we did not aim to conduct an exhaustive or systematic review. Our search terms and prioritization of materials identified may have missed or otherwise deprioritized relevant policies. This also means that we generally focused on regulations and guidance that expand upon, modernize, clarify, and interpret complex areas of law as opposed to the underlying, black letter law itself. As a result, our analysis is not a definitive legal analysis of SDOH data as health data. Finally, our understanding of the landscape is shaped by our interpretation of the materials we reviewed. Other researchers might reasonably reach different conclusions or emphasize different aspects of the same sources.

## Results

Current health care sector regulation of SDOH information can be grouped into 3 key purposes: (1) data standardization, interoperability, and exchange; (2) information privacy and security; and (3) responsible use in advanced analytics.

### Data Standardization, Interoperability, and Exchange

As attention to SDOH in the health care system has grown, so too has the need to document and share SDOH data. Shared data standards, such as vocabulary, support consistent formatting, and mutual understanding. They are critical for the smooth exchange of electronic health information (EHI) across various platforms, otherwise known as interoperability. Much of the development of social care vocabulary (ie, words and codes used to capture SDOH data) is driven by nonfederal stakeholders. In particular, the Gravity Project, a multistakeholder collaborative comprised of health care, health information technology (IT), community-based, federal and state agency, payer, academic, and patient/consumer advocacy sectors that launched in 2019, leads the creation of consensus-driven standards.^14^ Among other efforts, the Gravity Project was instrumental in recent expansions of *International Classification of Diseases*, *Tenth Revision* (*ICD-10*) Z codes representing SDOH-related health risks (eg, food insecurity).^15^ In contrast, federal and state policies have focused on creating the infrastructure and capacity to integrate social care data—described using that terminology–with clinical care data through the promotion of data standardization, interoperability, and exchange. Some of these policies are discussed in more detail later.

#### USCDI and the ONC Certification Program

The United States Core Data for Interoperability (USCDI) comprises a core set of data needed to support patient care. Maintained by the Office of the National Coordinator for Health Information Technology (ONC), the goal of the USCDI is to facilitate easy and accurate sharing of EHI across different health care systems and stakeholders by standardizing the way in which entities refer to the same patient observation.^16^ The USCDI supports the transition from, for example, an ecosystem in which one EHR describes a patient as “food insecure,” another documents the same patient observation as “nutrition insecure,” and a third uses the *ICD-10 Clinical Modification* Z code Z59.4 (standing for “lack of adequate food”) to an ecosystem in which all 3 EHRs use the appropriate Z code.

The current USCDI includes 4 SDOH data elements that relate to the use or purpose of SDOH data—that is, activities in clinical care: SDOH assessment, SDOH problems/health conditions, SDOH goals, and SDOH interventions.^17^ (Notably lacking from the current USCDI is an outcomes element—ie, a standardized way to capture measurable results of an SDOH intervention.) Each element is expressed using 1 or more designated, “applicable vocabulary standards” or coding systems.^17^ See Table 1.

**Table 1.** SDOH Information in the USCDI^17^^,^^19^

**Existing USCDI Data Class**	**Data Element**	**Applicable Vocabulary Standard(s)**
Assessment	SDOH assessment—Health professional’s conclusions and working assumptions, related to the conditions in which people live, learn, work, and play, which will guide treatment of the patient.	LOINC
Problems	SDOH problems/health concerns—social determinants of health-related concerns, conditions, or diagnoses.	SNOMED; *ICD*-10 Z codes
Goals	SDOH goals—Desired future states (eg, food security) for an identified social determinants of health-related health concern, condition, or diagnosis.	SNOMED; LOINC
Procedures	SDOH interventions—Actions or services to address an identified social determinants of health-related concern, condition, or diagnosis. Examples include but are not limited to education about food pantry program and referral to nonemergency medical transportation program.	SNOMED; CPT; HCPCS II

Abbreviations: ICD-10, International Classification of Diseases, Tenth Revision; SDOH, social determinants of health; USCDI, United States Core Data for Interoperability.

Beginning January 1, 2026, to be certified health IT, EHR, and other health IT software must be able to support USCDI Version 3.^16^^,^^18^ This means the technology must be able to exchange and utilize the SDOH data elements described earlier.^16^ More than 96% of hospitals and 78% of office-based physicians nationwide rely on certified health IT, suggesting expansive opportunity to standardize SDOH exchange in the near term.^16^ However, purchasers of certified health IT modules (eg, hospitals and physician offices) are encouraged—not mandated—to explore the use and incorporation of various functionalities. In practice, Z codes and other SDOH vocabulary remain underutilized in documentation.^19^^,^^20^

#### Information Resources to Support SDOH Data Exchange

In addition to USCDI reforms, federal regulators have furthered a commitment to ethical, robust, and effective SDOH data use and exchange through informational resources such as the 2023 SDOH Information Exchange Toolkit (the “Toolkit”) and the Social Determinants of Health Information Exchange Learning Forum (the “Learning Forum”).^21^

The Toolkit was created to “support conveners, facilitators, implementers, and the health IT community in the process of collaborative assessment, design, implementation, and governance needed to integrate information systems across sectors.”^22^ Specifically, the Toolkit identifies a series of foundational elements for information exchange activities and describes opportunities, challenges, and strategies for each.^22^ The elements recommended by ONC are: (1) community readiness and stewardship, (2) articulation of mission and purpose, (3) creation of underlying values and principles for information exchange, (4) consideration of policy levers and alignment with other relevant efforts, (5) establishment of an appropriate legal framework, (6) measurement and evaluation, (7) financing, (8) implementation support, and (9) technical infrastructure and data standards.^21^ The Learning Forum was designed to build on the work of the Toolkit by bringing together stakeholders to share lessons learned and promising practices from implementation.^23^

#### Care Coordination Infrastructure

There is significant variation in care coordination activities across states. At least 13 states have led, are leading, or are actively exploring investment in *unified statewide technical infrastructure development* for health care and social care data exchange and referrals between the sectors: Arizona, Colorado, Georgia, Massachusetts, Montana, North Carolina, New Hampshire, New Mexico, Oklahoma, Oregon, Pennsylvania, Tennessee, and Virginia. See Exhibit A. This means that the state has built or contracted with, or has indicated an intention to build or contract with (eg, through an official procurement request), technology-based solutions for information exchange between the health care and social care sectors.

A review of these efforts highlights several legal and policy choices that underpin infrastructure development.

- *Provider participation*: Provider participation is largely voluntary, although some states offer incentives to promote uptake. For example, in Arizona’s Community Cares referral system, both health care providers and community-based organizations may be eligible for financial incentives for participation.^24^
- *Target market*: Generally, the ability to integrate technology with Medicaid systems is a priority for the state; however, use of the platform may^24^ or may not^25^ be exclusive to Medicaid.
- *Directionality of information exchange*: States commonly prioritize bidirectional systems such as closed-loop referral systems that enable health care and social service providers to send referrals for services, confirm that referrals are received and acted upon, and track outcomes (see Exhibit A).
- *Integration with existing technical infrastructure*: States are taking different approaches regarding technology choices. In some states, such as North Carolina, a third-party technology solution is procured (ie, NCCARE 360).^26^ In some other jurisdictions, community-clinical care coordination is facilitated through existing HIE infrastructure. Some building out of existing infrastructure may be necessary, as well as the ability to integrate with other third-party platforms. In Washington, DC, for example, the Community Resource Information Exchange (CoRIE) Project connects health care and social services through the Washington, DC HIE.^27^ Access is not dependent on any single third-party social care platform; CoRIE is agnostic as to vendors such as Findhelp and Unite Us.^27^

California exemplifies an alternate approach to state investment in data exchange infrastructure. Rather than invest in technical infrastructure, the California Health and Human Services Agency has created a robust data exchange framework.^28^ The Data Exchange Framework (DxF) has 2 core components: (1) a single data-sharing agreement that all participants must sign; and (2) a common set of policies and procedures that govern the exchange of health and social services information.^28^ This approach is appealing for states reluctant to invest in a new statewide technology-based system because of preexisting infrastructure. In Maryland, for example, regulators rejected calls for a new closed-loop referral system because “many [managed care organizations] have already invested resources into their own [closed-loop referral systems]. It would take time, effort, and investment for these [organizations] to switch to a new system.”^29^

Finally, additional regulatory activity is underway at the state level in connection with evolving Medicaid flexibilities that allow for HRSN services and supports to be part of a beneficiary’s care. Some states have released detailed data exchange guidance for health care organizations and their social care vendors. In California, for example, the state has issued “comprehensive guidance” for Medicaid managed care plans conducting closed-loop referrals as part of the state’s HRSN demonstration waiver (CalAIM).^30^ In Michigan, in lieu of services policy to promote food and nutrition supports in the state’s Medicaid program includes a detailed section on data systems and data sharing between plans and providers.^31^ Commonalities across implementation guidance documents include attention to: (1) minimum data elements, (2) transmission standards (eg, relating to the security of member information), and (3) tracking and monitoring.^30^^,^^31^

Provider incentives may play an important role in promoting engagement. Washington, for example, enacted legislation requiring regulators to “evaluate incentive approaches and recommend funding options to increase the collection of Z codes on individual Medicaid claims, in accordance with standard billing guidance and regulations” (Revised Code of Washington § 74.09.880 [Health Equity—Primary Care Transformation—Goals] 2021).

### Information Privacy and Security

Health care information privacy and security currently receives heightened regulatory attention with a primary focus on improving interoperability and bolstering cybersecurity. Fewer regulatory reforms directly address the creation, use, and disclosure of social care information by health care organizations. Federal and state developments (or lack thereof) in areas such as Health Insurance Portability and Accountability Act (HIPAA) and closed-loop referral systems demonstrate how law and policy are grappling with these concerns.

#### HIPAA

HIPAA regulates uses and disclosures of a patient’s health care information by health care providers, health plans, and health care clearinghouses (“covered entities”), as well as related business associates (42 U.S.C. § 1320d et al).

HIPAA, its implementing regulations (45 C.F.R. Parts 160 and 164) and related guidance documents (see, eg, HIPAA Guidance Materials)^32^ rarely speak directly to how this complex and significant regulatory framework applies to social care entities or to the sharing of patient information between health care and social care partners. Amid hundreds of frequently asked questions and responses published by the U.S. Department of Health and Human Services (HHS), only 1 references social care providers. The guidance document addresses whether HIPAA permits a health care provider to share protected health information with a non–health care provider third party for purposes of continuity of care.^33^ And further, whether a health care provider can refer a “homeless patient to a social services agency, such as a housing provider, when doing so may reveal that the basis for eligibility is related to mental health.”^33^ HHS explains that “health care providers who believe that disclosures to certain social service entities are a necessary component of, or may help further, the individual’s health or mental health care may disclose the minimum necessary [protected health information] to such entities without the individual’s authorization.”^33^ The agency proposed to codify this guidance in a 2021 Notice of Proposed Rulemaking (NPRM) that was part of a department-wide “regulatory sprint to coordinated care.”^34^ However, a final rule has not yet been promulgated, leaving relatively little HIPAA-focused guidance in this area.

#### Information-Blocking Prohibitions and Exceptions

Federal rules on interoperability and information blocking, stemming from the 21st Century Cures Act, generally restrict interference with the access, use, and exchange of EHI (45 C.F.R. Part 171). Regulated entities include certified health IT developers but also health care providers, HIE, and health information networks (45 C.F.R. Part 171).

Of particular relevance to this review, advocates have raised concerns about the impact of information-blocking regulations on increased access to sensitive social care information—in particular, access to a clinician’s suspicions of or a patient’s disclosure pertaining to interpersonal violence by the perpetrator.^35^^,^^36^ Similar concerns arise for other SDOH data elements that may be uniquely vulnerable to misuse, such as immigration status,^13^ housing instability,^37^ financial insecurity,^37^ or a history of incarceration or other involvement with the justice system.^12^^,^^38^ Inappropriate disclosure of this information could expose individuals to negative actions such as immigration enforcement, eviction, employment discrimination, or increased surveillance.^12^^,^^13^^,^^34^^,^^37^^,^^38^ ONC has asserted that health care providers may rely on exceptions under the law for privacy and “to take appropriate steps to reduce abuse risks”;^39^^(p25838)^ however, because of the vagueness of the standard and other challenges translating policy to practice, the threat of unintended harm remains.^37^

#### Privacy and Security in Certified Health IT

As part of the process for adopting a recent version of USCDI for health IT certification criteria, which includes expanded inclusion of SDOH data elements, commenters expressly “urged that SDOH data be protected to ensure the privacy and security of the information.”^16^ To this end, some commenters urged ONC to require granular data-segmentation policies—requirements to sequester from capture, access, or view certain data elements that are perceived by a legal entity, institution, organization, or individual as being undesirable to share.^16^ Segmentation of sensitive medical records allows for more targeted consent and moves away from “all-or-nothing” approaches.

ONC declined to adopt new standards to control sensitive data, such as SDOH data, represented in USCDI-required functionalities.^16^ However, the agency was not outright dismissive of commenters’ concern, acknowledging in even the proposed rule that “the need to protect sensitive health information is foundational to a health equity by design principle not only to protect patient privacy, but also to mitigate the risk of any unintended negative impact on an individual resulting from the disclosure of sensitive health information.”^40^^(p23821)^ Regulators pointed to existing support for privacy and security labels in information workflows and created a new technical requirement that supports the HIPAA Privacy Rule’s right of an individual to request a restriction on sharing their health information with third parties.^16^^,^^40^ Specifically, patients (and their authorized representatives) must be able to use an internet-based method to request a restriction for any data expressed in the USCDI.^16^^,^^40^

#### State Law and Guidance on Information Privacy

In some cases, states have begun to step in to address gaps in the current frameworks surrounding privacy of SDOH data. For example, as of January 2026, legislative proposals explicitly targeting social care information privacy in closed-loop referral systems (CLRSs) have been introduced in at least 8 states: California (social care: data privacy, A.B. 1011, 2023–2024 Reg. Sess. [Cal. 2023]), Kansas (prohibiting governmental entities from sharing or transmitting social care information into a closed-loop referral system, S.B. 234, 2023–2024 Reg. Sess. [Kan. 2023]), Massachusetts (an act for the protection and privacy of social care information, H.378, 2025–2026 Reg. Sess. [Mass. 2025]), Nebraska (Social Care Information Privacy Act, L.B. 592, 1st Sess. [Neb. 2023]), New Hampshire (relative to a closed-loop referral system in the department of health and human services, S.B. 423, 2022 Reg. Sess. [N.H. 2022]), New Jersey (regulates use of social care information, A.4482, 2024–2025 Reg. Sess. [N.J. 2024]), Utah (Social Care Information Privacy Requirements, S.B. 130, 2023 Gen. Sess. [Utah 2023]), and West Virginia (Privacy of Social Care Information Act, H.B. 5271, 2024 Reg. Sess. [W. Va. 2024]). Several of the proposals are largely uniform. Among other policies, they limit (1) the ability of government agencies and their contractors to add an individual’s personally identifiable information or social care information to a closed-loop referral system without consent; (2) the ability of a participating service provider to access an individual’s information unless that individual has been referred to that organization and gives consent to that organization to access their information; and (3) the sale or licensing of individually identifiable social care information without explicit written consent. California and New Jersey are notable outliers, with legislation more narrowly focused on the third risk described earlier (social care: data privacy, A.B. 1011, 2023–2024 Reg. Sess. [Cal. 2023]; regulates use of social care information, A.4482, 2024–2025 Reg. Sess. [N.J. 2024]). In these 2 states, participating organizations are prohibited from using social care information stored in, or transmitted through, a CLRS “for any purpose or purposes other than the social care purpose or purposes for which that social care information was collected” (social care: data privacy, A.B. 1011, 2023–2024 Reg. Sess. [Cal. 2023]; regulates use of social care information, A.4482, 2024–2025 Reg. Sess. [N.J. 2024]).

Another approach is for states to fill knowledge gaps about legal information exchange left by federal privacy law. For example, California’s State Health Information Guidance (SHIG) leverages a multistakeholder collaboration to develop materials that clarify laws governing the use and disclosure of health information.^41^ SHIG Volume 2 “provides clarification of federal and state law targeting the sharing of health and social services information to support the coordination of treatment/care and services related to food and nutrition insecurity.”^42^

### Advanced Analytics

The third target of regulation discussed in this article is advanced analytics—that is, the use of predictive modeling, machine learning algorithms, deep learning, business process automation, and other statistical methods to derive insights. Not only is advanced analytics a topic of growing regulatory scrutiny in the health care sector^43^ due to exposure of biased care coordination algorithms^44^ and reports of insurers using algorithms to deny care,^45^ but there is also increasing interest in using SDOH data to improve models that predict health care utilization, health care costs, and even HRSN.^46^ For example, artificial intelligence (AI) can be used to inform decision-making on cardiovascular disease risk.^47^ Researchers have used socioeconomic indicators to predict the incidence of hypertension^48^ and environmental risk has been used to predict hospital readmission following an initial cardiovascular event.^49^ Recent HHS regulation of decision support technology and algorithms in health care show emerging approaches to balancing these concerns.

#### Electronic Decision Support Interventions and Certified Health IT

In October 2023, the Biden Administration directed HHS to prepare a strategy for the responsible deployment and use of AI in health care.^50^ The resulting regulation seeks to ensure that certified health IT modules “reflect an array of contemporary functionalities, support data elements important to health equity, and enable the transparent use of predictive models and algorithms to aid decision-making in health care.”^16^^(p1196)^

The final rule differentiates between “predictive decision support interventions (DSIs)”—“technology that supports decision-making based on algorithms or models that derive relationships from training data and then produce an output that results in prediction, classification, recommendation, evaluation, or analysis”—and “evidence-based DSIs”—which are nonpredictive interventions.^16^ Among other policies, the final rule requires “source attribute transparency.” Users of health IT modules certified to the DSI criterion must have access to information about whether patient demographic, SDOH, or health assessment data are used in the DSIs.^16^ This measure is important because users are better able to understand the nature of a module, identify potential inherent biases, and determine how best to use the technology with specific patient populations. The burden ultimately falls on providers to evaluate these data as part of decision-making regarding the use of any DSI.

#### Section 1557 Nondiscrimination Rules

Alongside ONC’s efforts, HHS OCR has established more extensive enforcement authority over advanced analytics. Section 1557 of the Affordable Care Act prohibits discrimination on the basis of race, color, national origin, sex, age, or disability in a range of health programs and activities (42 U.S.C. § 18116). Although the regulatory landscape for Section 1557 is in flux, with ongoing litigation and administrative changes affecting the scope and durability of protections over time,^51^ the Biden-era Section 1557 rulemaking presents an interesting case study in the use of nondiscrimination protections to regulate data use in advanced analytics. The Biden Administration introduced explicit protections against discrimination through the use of “patient care decision support tools.”^52^^(p37642)^ Patient care decision support tools are further defined as “any automated or nonautomated tool, mechanism, method, technology, or combination thereof used by a covered entity to support clinical decision-making in its health programs or activities.”^52^^(p37695)^ The rule clarifies that covered tools include those used to assess health status, recommend care decisions, and/or conduct utilization management.^52^

Commenters expressed concern that the proposed regulation may unintentionally limit the ability of health care organizations to reduce health disparities. Some commenters explained that they invest in SDOH and other activities targeted to members of a specific, defined population (eg, high-risk pregnancies, individuals transitioning from incarceration to the community, and individuals living with specific chronic conditions).^51^ Another noted that, “if health plans are required to provide services that address chronic care, social determinants of care, or other similar programs ‘equally’ to all enrollees rather than ‘equitably’ target services to those in need based on health or socioeconomic condition, plans will be limited in their ability to provide appropriate services and scale and sustain these programs.”^52^^(p37617)^

In its response, OCR clarified that the final rule does not prohibit programs designed to improve health outcomes for specific populations, as long as the programs do not discriminate based on a protected basis.^52^ OCR further highlighted that SDOH data (eg, income level or zip code) may actually serve to identify individuals in a manner that does not violate Section 1557.^52^

#### State Regulation

Although states are increasingly regulating the use of AI for advanced analytics in health care, recent state legislation and legislative proposals do not explicitly address or otherwise focus on the incorporation of SDOH data into AI models. Moreover, recent executive action by the Trump Administration, EO 14365, aims to establish a national AI framework and challenges the role of states in AI regulation.^53^

## Discussion

The foregoing review of social care information regulation in the health care sector highlights both opportunities and challenges that must be carefully navigated to advance effective and equitable policy. Importantly, federal and state authorities are actively wrestling with several consequential regulatory design questions, including (1) whether to treat SDOH data created, collected, maintained, and used in or by health care settings in the same manner as any other type of health information; (2) appropriate levers to promote adoption of desired policies and achievement of desired outcomes; and (3) the rightsized role of the federal government and states. These policy crossroads speak to the importance of futureproofing regulation—that is, cultivating a regulatory environment for SDOH data collected, used, and exchanged by the health care sector that is not only responsive to the current realities of the field but also responsive to rapid evolution and resilient to future challenges.

### SDOH Data Exceptionalism

A recurring and critical tension centers on whether to treat SDOH data created, collected, maintained, and used in or by health care settings as any other type of health care data or with exceptionalism—specifically, heightened protections. An argument in favor of a separate regime is the fact that some populations that have historically been subject to discrimination may identify a wide range of social care information as particularly sensitive. Examples include immigration status, housing instability, a history of criminal-legal involvement, and substance use. On the other hand, “sensitive data” is overall a dynamic and individualized determination. Pregnancy status is far more sensitive for individuals in states hostile to abortion than individuals in states that are not, for example, as is one’s HIV status in states with HIV criminalization laws. These are clinical examples, but they underscore that the sensitivity of data is often context dependent. In this way, SDOH data and other types of health care data share important similarities and warrant similar treatment.

At least at the federal level, regulators have at times taken a balanced approach by discretely considering SDOH data as part of—versus parallel to—core opportunities for health care information regulation. We see this, for example, with HIPAA, information blocking, and even AI rulemaking activities. This degree of integration ensures that SDOH data is considered in important rulemaking and other cutting-edge health care information initiatives. This approach also avoids the creation of duplicative systems with associated increases in resource investment and compliance burden.

The balanced approach also offers several practical steps for health care organizations and their social care services partners. First, integration implementers can explicitly classify in their own policies which SDOH elements are treated as “heightened-sensitivity” data and under what circumstances. Second, organizations can incorporate questions about context-specific sensitivity—such as immigration enforcement risk, housing precarity, or criminal-legal involvement—into consent conversations, staff training, and decisions about what is documented in the EHR or shared with third-party platforms. Finally, vendors and implementers of care coordination and referral tools can design configurable controls (eg, role-based access or data segmentation flags) so that local partners can operationalize more protective practices where they are warranted.

### Appropriate Regulatory Levers

Another design question concerns the choice of regulatory levers. Policymakers are testing whether to foster adoption of SDOH data practices through permissions, incentives (eg, carrots and sticks), strict mandates, or a blend of the 3. These different regulatory levers map onto day-to-day choices by stakeholders, including health plans, delivery systems, and HRSN service providers.

The decentralized and uneven integration of social care services and supports into health care across US communities complicate uniform, prescriptive regulation. Tensions are heightened where SDOH data collection, use, and exchange adds a layer of complexity onto preexisting frictions in health information policy relating, for example, to interoperability (eg, where health care and social care organizations use different information systems) and workflow burden (eg, where special consents are required for SDOH data use and exchange, individualized assessments of the sensitivity of data). At the same time, without assertive federal and state intervention, barriers to the meaningful use and protection of SDOH data will linger—potentially perpetuating inequities in both health outcomes and data privacy. Especially in the absence of more aligned SDOH reimbursement policy, incentives, such as Medicaid agency payments for providers to participate in SDOH information exchange, play a critical role at this juncture. Notably, this reality reflects, in many ways, the historical role of incentives in promoting the adoption and meaningful use of EHRs and EHR interoperability.^54^^,^^55^ In-kind supports, such as technical infrastructure, the identification and dissemination of best practices, and readily adaptable risk management solutions (eg, privacy and security policies) are also important as they help lower the costs of adoption for implementers.

### Jurisdictional Balance

The question of jurisdiction—the rightsized role of the federal government and states**—**further complicates the regulatory picture. By and large, federal regulation creates an enabling environment for innovation upon which states are building.

This division makes sense for reasons including, as noted elsewhere, that states are experimenting with HRSN benefits in Medicaid in different ways and to different degrees. States that are building CLRS or expanding HRSN benefits can embed baseline protections in procurement documents, participation agreements, and technical specifications—such as prohibitions on secondary uses of social care data, minimum consent standards, and requirements to offer patients meaningful choices about what is shared with whom.

However, we are also entrenching ourselves in a scenario in which SDOH data exchange is more clearly part of a new standard of care in some states than in others, and in which SDOH data are more protected in some states than others. As described in our regulatory review, not all states are grappling with best practices in meaningful ways or even at all. To prevent further exacerbating inequities between states, states should proactively address critical consumer protections *and* any federal floor must provide a solid foundation.

Local actors in states without explicit regulation also have a role to play. They can look to emerging state models and federal guidance as de facto best practices, rather than waiting for formal mandates, thereby reducing variation in protections experienced by patients who move across state lines or between health systems.

Each of these design tensions—whether in defining the nature of SDOH data, selecting effective levers for change, or navigating federal-state dynamics—underscore the fluidity and complexity of regulatory efforts and presents a moving target that risks outpacing static regulatory approaches. We require frameworks that anticipate continuing changes in technology, health care delivery, and societal values. Policymakers will need to address not only how SDOH data are protected and exchanged today but also how future uses—intended or otherwise—may affect diverse populations. This demands ongoing dialogue among stakeholders, iterative rulemaking, and mechanisms for assessing the real-world impacts of regulatory choices. For example, future research will be essential to monitor and understand the effects of different privacy models and the outcomes for patients and communities most affected by new data practices. Research should also explore how evolving technologies, such as AI, intersect with SDOH data, as well as methods for measuring the effectiveness of regulatory safeguards in minimizing harm and promoting equity.

## Conclusion

Driven by evolving health care policy, SDOH information is increasingly working its way into EHRs, HIEs, care coordination platforms, payer databases, and patient applications. Regulation of social care data as health care data is also multifaceted, evolving, and consequential as both the free flow of that information between systems and safeguards to push back against potentially harmful applications thereof are becoming more pressing priorities. With the trajectory set to continue, federal and state policymakers should proactively shore up the regulatory frameworks that govern SDOH data as health information and design them with an eye toward adaptability, equity, and the lived realities of the populations most affected.

In the near term, health care organizations, payers, and social care partners do not need to wait for perfect regulatory clarity to take action. They can adopt internal policies that treat social risk information as sensitive by default; invest in transparent, patient-centered consent and communication practices; and insist, through contracting and procurement, that technology vendors support privacy-preserving configuration of SDOH data elements and AI-enabled tools. By doing so, practitioners can help build the practical norms and evidence base that will, in turn, inform the next generation of federal and state law and policy governing SDOH data as health care data.

## Author Contributions

All the authors have each contributed materially to this manuscript.

## Disclosures

The authors have nothing to disclose.

## Funding

The authors received no funding for this research.

CE Quiz

## Supplementary Material

Supplemental Material

## Appendix

**Exhibit A.** Analysis of State-led Technical Infrastructure for Health and Social Care Information Exchange

**State**	**Description**	**Materials Indicating State Leadership in Technical Infrastructure: Document, Website, or Web Page Title**	**Materials Indicating State Leadership in Technical Infrastructure Development: Link to Document, Website, or Web Page**
Alabama	None identified		
Alaska	None identified		
Arizona	“AHCCCS has partnered with Contexture and Unite Us to provide CommunityCares, Arizona’s Statewide Closed-Loop Referral System ... CommunityCares is a free tool available to help AHCCCS health care providers and community-based organizations quickly and efficiently screen and refer members for Health-Related Social Needs, also known as Social Determinants of Health.”	CommunityCares Arizona’s Statewide Closed-Loop Referral System	https://www.azahcccs.gov/communitycares
Arkansas	Nothing identified		
California	Nothing identified		
Colorado	“CoSHIE is a network to securely share physical, behavioral, and social health information between providers involved in whole-person care.”	Colorado Social Health Information Exchange (CoSHIE)	https://oehi.colorado.gov/SHIE
Connecticut	None identified		
District of Columbia	Aims of the CoRIE initiative include to “connect health and social service providers using the DC HIE without requiring a single technology platform”	Community Resource Information Exchange Project (CoRIE)	https://crispdc.org/wp-content/uploads/2021/09/CoRIE-one-pager-3.04.21.pdf
Delaware	None identified		
Florida	None identified		
Georgia	“GeorgiaUnify® is an innovative social care integration platform that leverages [Georgia’s state-designated health information exchange] network and technology platform to allow health systems, public health agencies, providers, and hospitals to exchange information and coordinate care with a wide range of social care organizations.”	Georgia Unify	https://www.gahin.org/products-services/georgiaunify
Hawaii	None identified		
Idaho	The Idaho Health Data Exchange (IHDE) collaborated with Findhelp to provide a safe, secure, and effective platform for IHDE users to connect patients with social services. However, the State terminated its contract with IHDE in 2024.	Findhelp.org Partners Notice of Termination for Contract No. RC072900	https://www.findhelpidaho.org/partners https://idahocapitalsun.com/wp-content/uploads/2024/05/IHDE-Termination-Letter.pdf
Illinois	None identified		
Indiana	None identified		
Iowa	None identified		
Kansas	None identified		
Kentucky	None identified		
Louisiana	None identified		
Maine	None identified		
Maryland	None identified		
Massachusetts	The State’s Capital Investment Plan includes funding to “implement a standardized, statewide electronic Health Related Social Needs (HRSN) referral platform to allow for efficient, closed loop referral and communication between MassHealth providers and social service organizations delivering services to MassHealth members with HRSNs.”	I432 MassHealth Elect. Health-Related Social Needs (HRSN) Referral Platform	https://budget.digital.mass.gov/capital/fy24/beneficiary-agency/health-and-human-services/exec-office-of-health-and-human-services/i432/
Michigan	None identified		
Minnesota	None identified		
Mississippi	None identified		
Missouri	None identified		
Montana	“CONNECT is a bidirectional referral network that allows client contact information to be sent between service providers. The secure web-based system is available at no cost to approved organizations that make client referrals.”	CONNECT Referral System: Connecting Service Providers Throughout Montana	https://dphhs.mt.gov/publichealth/ConnectMT/CONNECTReferralSystem
Nebraska	None identified		
Nevada	None identified		
New Hampshire	“This Request for Proposal (RFP) is published to solicit proposals for a closed loop referral solution, meaning a system that will enhance care coordination for those clients providing informed consent by enabling health care and community service providers to connect on a single statewide technology platform, in accordance with RSA 126-A:4.”	RFP-2024-OCOM-02-CLOSE: Closed Loop Referral	https://www.dhhs.nh.gov/news-and-media/rfp-2024-ocom-02-close-closed-loop-referral
New Jersey	None identified		
New Mexico	“This Request for Quotes (RFQ) is intended to invite vendors to submit their best and most competitive quotes for providing a comprehensive, efficient, and user-friendly CLRS that will meet the needs of the New Mexico Human Services Department (HSD) and partner State agencies, Community Based Organizations (CBO), health care professionals, patients, and communities.”	Closed Loop Referral Service Request for Quotes	https://www.hca.nm.gov/wp-content/uploads/CLRS_RFQ_101823.pdf
New York	None identified (delegated to Social Care Networks as part of 1115 Medicaid waiver)		
North Carolina	“The network bridges gaps in a fragmented health and human services system by connecting providers and organizations across sectors through a shared technology platform.”	NCCARE360	https://nccare360.org/
North Dakota	None identified		
Ohio	None identified		
Oklahoma	In 2024, Findhelp was awarded a contract with the Oklahoma Health Care Authority “to develop and maintain a Closed Loop Electronic Referral System (CLERS) to coordinate electronic referrals between organizations for the purpose of addressing the social drivers of health (SDoH) of Oklahomans in need.”	Findhelp Announces Strategic Partnership with the Oklahoma Health Care Authority to Launch Statewide Closed Loop Electronic Referral System	https://www.prnewswire.com/news-releases/findhelp-announces-strategic-partnership-with-the-oklahoma-health-care-authority-to-launch-statewide-closed-loop-electronic-referral-system-302075014.html
Oregon	“OHA plans to procure CIE for OHP Open Card (also known as fee-for-service) to meet the closed loop referral requirements for HRSN services and for OHA and Oregon Department of Human Services (ODHS) staff who need access to support the waiver.”	Community Information Exchange to Support Oregon’s 1115 Medicaid Waiver	https://www.oregon.gov/oha/HPA/OHIT/Documents/CIEtoSupportOregons1115MedicaidWaiverInformationalBrief.pdf
Pennsylvania	“PA Navigate connects Pennsylvanians with health and social care services in their local community.”	PA Navigate	https://pa-navigate.org/
Rhode Island	None identified		
South Carolina	None identified		
South Dakota	None identified		
Tennessee	“TennCare selected a Closed Loop Referral System (CLRS) vendor, Findhelp, through a competitive procurement process. The TennCare CLRS has been branded as ‘Tennessee Community Compass’.”	TN Division of TennCare, Health Starts	https://www.tn.gov/tenncare/providers/programs-and-facilities/health-starts.html
Texas	None identified		
Utah	None identified		
Vermont	None identified		
Virginia	“Governor Ralph Northam . . . announced that Virginia will allocate $10M in federal Coronavirus Aid, Relief, and Economic Security (CARES) Act funding to create Unite Virginia, a statewide technology platform designed to connect vulnerable Virginians to health and social services.”	Virginia to Partner with Unite Us to Create Statewide Infrastructure Connecting Health and Social Services	https://uniteus.com/press/virginia-to-partner-with-unite-us-to-create-statewide-infrastructure-connecting-health-and-social-services-3/
Washington	None identified		
West Virginia	None identified		
Wisconsin	None identified		
Wyoming	None identified		
