# Supplementary material for: SDOH Data as Health Care Data: The Multifaceted, Evolving, and Consequential Role of Law and Policy
Source: Adv Health Inf Sci Pract. 2026 Sep 1;2(2):eah.000000006. doi: 10.63116/AH.000000006 (PMC13598768; doi:10.63116/AH.000000006)
Supplement: Supplementary file 1 [file Supplemental_Materials_Exhibit_A.docx]

Exhibit A. Analysis of State-led Technical Infrastructure for Health and Social Care Information Exchange

| State | Description | Materials Indicating State Leadership in Technical Infrastructure: Document or Webpage Title | Materials Indicating State Leadership in Technical Infrastructure Development: Link to Document or Website |
| --- | --- | --- | --- |
| Alabama | None identified | | |
| Alaska | None identified | | |
| Arizona | “AHCCCS has partnered with Contexture and Unite Us to provide CommunityCares, Arizona’s Statewide Closed-Loop Referral System. . . . CommunityCares is a free tool available to help AHCCCS health care providers and community-based organizations quickly and efficiently screen and refer members for Health-Related Social Needs, also known as Social Determinants of Health.” | Arizona Health Care Cost Containment System | https://www.azahcccs.gov/communitycares |
| Arkansas | Nothing identified | | |
| California | Nothing identified | | |
| Colorado | “CoSHIE is a network to securely share physical, behavioral, and social health information between providers involved in whole-person care.” | Colorado Social Health Information Exchange (CoSHIE) | https://oehi.colorado.gov/SHIE |
| Connecticut | None identified | | |
| District of Columbia | Aims of the CoRIE initiative include to “connect health and social service providers using the DC HIE without requiring a single technology platform” | Community Resource Information Exchange (CoRIE) | https://crispdc.org/wp-content/uploads/2021/09/CoRIE-one-pager-3.04.21.pdf |
| Delaware | None identified | | |
| Florida | None identified | | |
| Georgia | “GeorgiaUnify® is an innovative social care integration platform that leverages [Georgia’s state-designated health information exchange] network and technology platform to allow health systems, public health agencies, providers and hospitals to exchange information and coordinate care with a wide range of social care organizations.” | Georgia Unify | https://www.gahin.org/products-services/georgiaunify |
| Hawaii | None identified | | |
| Idaho | “[The Idaho Health Data Exchange (IDHE)] is collaborating with Findhelp to provide a safe, secure, and effective platform for IHDE users to connect patients with social services.” However, the State terminated its contract with IDHE in 2024. | Idaho Health Data Exchange: Findhelp | https://idahohde.org/aunt-bertha-social-determinants-of-health-tool/ |
| Illinois | None identified | | |
| Indiana | None identified | | |
| Iowa | None identified | | |
| Kansas | None identified | | |
| Kentucky | None identified | | |
| Louisiana | None identified | | |
| Maine | None identified | | |
| Maryland | None identified | | |
| Massachusetts | The State’s Capital Investment Plan includes funding to “implement a standardized, statewide electronic Health Related Social Needs (HRSN) referral platform to allow for efficient, closed loop referral and communication between MassHealth providers and Social Service Organizations delivering services to MassHealth members with HRSNs.” | MassHealth Elect. Health-Related Social Needs (HRSN) Referral Platform | https://budget.digital.mass.gov/capital/fy24/beneficiary-agency/health-and-human-services/exec-office-of-health-and-human-services/i432/ |
| Michigan | None identified | | |
| Minnesota | None identified | | |
| Mississippi | None identified | | |
| Missouri | None identified | | |
| Montana | “CONNECT is a bidirectional referral network that allows client contact information to be sent between service providers. The secure web-based system is available at no cost to approved organizations that make client referrals.” | CONNECT Referral System: Connecting Service Providers Throughout Montana | https://dphhs.mt.gov/publichealth/ConnectMT/CONNECTReferralSystem |
| Nebraska | None identified | | |
| Nevada | None identified | | |
| New Hampshire | “This Request for Proposal (RFP) is published to solicit proposals for a closed loop referral solution, meaning a system that will enhance care coordination for those clients providing informed consent by enabling health care and community service providers to connect on a single statewide technology platform, in accordance with RSA 126-A:4.” | RFP-2024-OCOM-02-CLOSE: Closed Loop Referral | https://www.dhhs.nh.gov/news-and-media/rfp-2024-ocom-02-close-closed-loop-referral |
| New Jersey | None identified | | |
| New Mexico | “This Request for Quotes (RFQ) is intended to invite vendors to submit their best and most competitive quotes for providing a comprehensive, efficient, and user-friendly CLRS that will meet the needs of the New Mexico Human Services Department (HSD) and partner State agencies, Community Based Organizations (CBO), health care professionals, patients, and communities.” | Closed Loop Referral Service Request for Quotes | https://www.hca.nm.gov/wp-content/uploads/CLRS_RFQ_101823.pdf |
| New York | None identified (delegated to Social Care Networks as part of 1115 Medicaid waiver) | | |
| North Carolina | “**The network bridges gaps in a fragmented health and human services system by connecting providers and organizations across sectors through a shared technology platform.”** | NCCARE360 | https://nccare360.org/ |
| North Dakota | None identified | | |
| Ohio | None identified | | |
| Oklahoma | In 2024, Findhelp was awarded a contract with the Oklahoma Health Care Authority “to develop and maintain a Closed Loop Electronic Referral System (CLERS) to coordinate electronic referrals between organizations for the purpose of addressing the social drivers of health (SDoH) of Oklahomans in need.” | Findhelp Announces Strategic Partnership with the Oklahoma Health Care Authority to Launch Statewide Closed Loop Electronic Referral System | https://www.prnewswire.com/news-releases/findhelp-announces-strategic-partnership-with-the-oklahoma-health-care-authority-to-launch-statewide-closed-loop-electronic-referral-system-302075014.html |
| Oregon | “OHA plans to procure CIE for OHP Open Card (also known as fee-for-service) to meet the closed loop referral requirements for HRSN services and for OHA and Oregon Department of Human Services (ODHS) staff who need access to support the waiver.” | Community Information Exchange to Support Oregon’s 1115 Medicaid Waiver | https://www.oregon.gov/oha/HPA/OHIT/Documents/CIEtoSupportOregons1115MedicaidWaiverInformationalBrief.pdf |
| Pennsylvania | “PA Navigate connects Pennsylvanians with health and social care services in their local community.” | PA Navigate | https://pa-navigate.org/ |
| Rhode Island | None identified | | |
| South Carolina | None identified | | |
| South Dakota | None identified | | |
| Tennessee | “TennCare selected a Closed Loop Referral System (CLRS) vendor, Findhelp, through a competitive procurement process. The TennCare CLRS has been branded as “Tennessee Community Compass”.” | TN Division of TennCare, Health Starts | https://www.tn.gov/tenncare/providers/programs-and-facilities/health-starts.html |
| Texas | None identified | | |
| Utah | None identified | | |
| Vermont | None identified | | |
| Virginia | “Governor Ralph Northam. . .announced that Virginia will allocate $10M in federal Coronavirus Aid, Relief, and Economic Security (CARES) Act funding to create Unite Virginia, a statewide technology platform designed to connect vulnerable Virginians to health and social services.” | Virginia to Partner with Unite Us to Create Statewide Infrastructure Connecting Health and Social Services | https://uniteus.com/press/virginia-to-partner-with-unite-us-to-create-statewide-infrastructure-connecting-health-and-social-services-3/ |
| Washington | None identified | | |
| West Virginia | None identified | | |
| Wisconsin | None identified | | |
| Wyoming | None identified | | |
